# Population pharmacokinetics of nintedanib, an inhibitor of tyrosine kinases, in patients with non-small cell lung cancer or idiopathic pulmonary fibrosis

**DOI:** 10.1007/s00280-017-3452-0

**Published:** 2017-11-08

**Authors:** Ulrike Schmid, Karl-Heinz Liesenfeld, Angele Fleury, Claudia Dallinger, Matthias Freiwald

**Affiliations:** Department of Translational Medicine and Clinical Pharmacology, Boehringer Ingelheim Pharma GmbH & Co. KG, Birkendorfer Strasse 65, 88397 Biberach an der Riss, Germany

**Keywords:** Covariates, Idiopathic pulmonary fibrosis, Nintedanib, Non-small cell lung cancer, Population pharmacokinetics

## Abstract

**Purpose:**

A population pharmacokinetic model was developed for nintedanib in patients with non-small cell lung cancer (NSCLC) or idiopathic pulmonary fibrosis (IPF). The effects of intrinsic and extrinsic patient factors on exposure of nintedanib and its main metabolite BIBF 1202 were studied.

**Methods:**

Data from 1191 patients with NSCLC (*n* = 849) or IPF (*n* = 342) treated with oral nintedanib (once- or twice-daily, dose range 50–250 mg) in 4 Phase II or III studies were combined. Plasma concentrations of nintedanib (*n* = 5611) and BIBF 1202 (*n* = 5376) were analyzed using non-linear mixed-effects modeling.

**Results:**

Pharmacokinetics of nintedanib were described by a one-compartment model with linear elimination, first-order absorption, and absorption lag time. For a typical patient, the absorption rate was 0.0827 h^−1^, apparent total clearance was 897 L/h, apparent volume of distribution at steady state was 465 L, and lag time was 25 min. Age, weight, smoking, and Asian race were statistically significant covariates influencing nintedanib exposure, but no individual covariate at extreme values (5th and 95th percentiles of baseline values for continuous covariates) resulted in a change of more than 33% relative to a typical patient. Pharmacokinetics and covariate effects for BIBF 1202 were similar to nintedanib. Mild or moderate renal impairment and mild hepatic impairment (classified by transaminase or bilirubin increase above the upper limit of normal) or underlying disease had no significant effects on nintedanib pharmacokinetics.

**Conclusions:**

This model adequately described the pharmacokinetic profile of nintedanib in NSCLC and IPF populations and can be used for simulations exploring covariate effects and exposure–response analyses.

**Electronic supplementary material:**

The online version of this article (doi:10.1007/s00280-017-3452-0) contains supplementary material, which is available to authorized users.

## Introduction

Nintedanib is a kinase inhibitor targeting VEGFR (vascular endothelial growth factor receptor) 1, 2, and 3, PDGFR (platelet-derived growth factor receptor) α and β, and FGFR (fibroblast growth factor receptor) 1, 2, and 3 [1–3]. These kinase receptors have been shown to play an important role not only in angiogenesis but also in tumor growth and metastasis [4]. Nintedanib has shown clinical efficacy in non-small cell lung cancer (NSCLC) [5, 6] and in 2014 was approved in Europe in combination with docetaxel for use in adult patients with locally advanced, metastatic, or locally recurrent NSCLC of adenocarcinoma tumor histology, after first-line chemotherapy [3, 7].

VEGFR, PDGFR, and FGFR have also been shown to participate in the mechanisms underlying the disease of idiopathic pulmonary fibrosis (IPF) [8–10]. Therefore, the specific kinase inhibitory profile of nintedanib was considered to be a potentially beneficial therapy for IPF, a chronic, devastating disease of unknown etiology characterized by progressive fibrotic destruction of the lung. Based on results of one Phase II trial (TOMORROW) [11] and two identically designed Phase III trials (INPULSIS-1 and INPULSIS-2), which demonstrated a significant reduction in the rate of decline in forced vital capacity over a 52-week treatment period [12], nintedanib is approved for the treatment of IPF in various countries, including the US, Canada, Japan, and Europe [13, 14].

In patients with solid tumors, peak plasma concentrations of nintedanib occur approximately 2–4 h after oral dosing [15–17]. Nintedanib is typically administered in twice-daily doses, with steady state achieved within 7 days of twice-daily dosing [18, 19]. The total and peak systemic exposures are dose-proportional and absolute bioavailability of nintedanib is about 5% [19]. The total fraction of nintedanib and its metabolites absorbed is estimated to be much higher than the absolute bioavailability of nintedanib alone (23 versus 5%, [19]), suggesting a high intestinal and/or hepatic first-pass metabolism. There is moderate-to-high inter-subject variability for the area under the plasma concentration–time curve for the dosing interval at steady state (AUC_τ,ss_) [15, 16, 20], and the geometric mean terminal elimination half-life is about 10–15 h [16, 19]. In a Phase I trial, food increased nintedanib exposure (AUC_0−∞_) and maximum plasma concentrations (*C*_max_) by 20% and absorption was delayed [17]. In Phase II and III trials, nintedanib was generally administered with food and current prescribing guidelines recommended that it should be taken with food [13].

Nintedanib is rapidly metabolised, primarily via ester cleavage, resulting in the formation of the free acid moiety (BIBF 1202) that is subsequently glucuronidated by hepatic and intestinal uridine 5′-diphospho-glucuronosyltransferase (UGT) 1A1 (UGT1A1) enzymes to form BIBF 1202 glucuronide and excreted in the feces (about 94% of the dose) [16]. Less than 1% is eliminated in urine. Drug interactions based on cytochrome P-450 pathways are not expected. Nintedanib is a substrate of P-glycoprotein (P-gp) and co-administration of potent P-gp modulators is associated with small-to-moderate changes in exposure (e.g., co-administration with 400 mg ketoconazole [potent inhibitor], increased nintedanib exposure by 60%, whereas co-administration of nintedanib with 600 mg rifampicin [potent inducer] decreased nintedanib exposure by 50% [13, 21, 22]).

Although the principal metabolite BIBF 1202 shows pharmacological activity at some of the target receptors, in vivo cellular activity indicates a substantially lower potency compared to nintedanib (~ 9–10-fold based on VEGF or bFGF stimulation of human umbilical vascular endothelial cells; 265- and 607-fold based on PDGFRα and PDGFRβ stimulation of primary lung fibroblasts, respectively) (Boehringer Ingelheim unpublished data, [14]). BIBF 1202 was not effective in mouse xenograft models [7], suggesting that BIBF 1202 plasma levels themselves are unlikely to contribute to the clinical effects of nintedanib in vivo. BIBF 1202 glucuronide did not show in vitro activity at the target receptors and is a non-reactive glucuronide [16].

The primary objective of this population pharmacokinetic analysis was to characterize the pharmacokinetic plasma profile of nintedanib in the target populations of NSCLC and IPF patients and to evaluate the effect of selected intrinsic and extrinsic factors on exposure of nintedanib. In view of its lower in vivo potency compared with nintedanib, a secondary objective was to characterize plasma exposure of the metabolite BIBF 1202 and to assess the influence of intrinsic and extrinsic factors on its exposure.

## Subjects and methods

### Trials included in the analysis

This population pharmacokinetic analysis pooled data from nintedanib treatment in four clinical studies; 1 Phase II (*n* = 73) [23] and two Phase III trials in patients with NSCLC (LUME-Lung 1 (*n* = 652) [5] and LUME-Lung 2 (*n* = 347) [6]) and one Phase II trial (TOMORROW, *n* = 343) in patients with IPF [11] (data from IPF Phase III trials were not available at time of analysis). An overview of these studies is provided in Online Resource Table S1. In the NSCLC Phase II trial [23], patients with locally advanced or metastatic relapsed NSCLC in whom first- or second line platinum-based chemotherapy had failed and were randomized to nintedanib 150 or 250 mg twice-daily. Treatment continued until disease progression or intolerable toxicity. In the NSCLC Phase III trials, nintedanib 200 mg twice-daily or placebo was given in repeated 21 day treatment cycles in combination with single infusions of docetaxel 75 mg/m^2^ (LUME-Lung 1 trial [5]) or pemetrexed 500 mg/m^2^ (LUME-Lung 2 trial [6]), administered on day 1 of each cycle. Treatment cycles were continued until disease progression or intolerable toxicity. In the Phase II trial in patients with IPF [11], patients were randomized to a 52-week treatment period with one of the four doses of nintedanib (50 mg once or twice-daily, 100, 150 mg twice-daily) or placebo. In all four trials, dose reductions and interruptions were allowed to manage adverse events. All studies were conducted in accordance with the Declaration of Helsinki and with approval of the local ethics committees. Written informed consent was obtained from all patients before study entry.

### Pharmacokinetic sampling and bioanalytical assays

Blood sampling schema for measurement of nintedanib plasma concentrations varied. For all trials, at least two pre-dose and two post-dose blood samples were scheduled during treatment (see Online Resource Table S1). In addition, more extensive sampling was performed in the two Phase II trials [11, 23]. Nintedanib and BIBF 1202 plasma concentrations were analyzed by validated liquid chromatography–mass spectrometry (HPLC–MS/MS) methods [16]. The calibration curves covered the range 0.5–500 ng/mL for nintedanib and 1–1000 ng/mL for BIBF 1202 in undiluted plasma samples and were linear over this range.

### Data analysis

The population pharmacokinetic analysis was performed using non-linear mixed-effects modeling techniques (NONMEM, version VI 2.0, ICON Development Solutions, Ellicott City, Maryland, USA). The first-order conditional estimation algorithm with interaction was used. Data processing, diagnostic plots, and calculation of descriptive statistics were performed using R version 2.12.1 (http://www.rproject.org) and SAS version 9.2 (SAS Institute Inc, Cary, NC, USA). Bootstrap analysis was performed using Perl-speaks-NONMEM, version 3.5.3 [24, 25].

### Base model development

A sequential approach was used for population pharmacokinetic model development. First, the base model was developed for nintedanib by (1) investigating the compartmental model structure including evaluation regarding dose linearity as well as trial effects and (2) investigating the stochastic model. Inter-individual variability (IIV, *η*) and inter-occasion variability (IOV, κ) were modeled using exponential random effect models. For testing IOV, each visit was defined as one occasion. IIV, IOV, and residual (unexplained) variability (*ε*) were assumed to be symmetrically distributed around 0 with variances *ω*_IIV_^2^, *ω*_IOV_^2^, and *σ* ^2^, respectively. Model selection was guided by change in objective function values (OFV), identifiability of parameters and precision of parameter estimates, correlation between the estimates of fixed-effect parameters, numerical stability, ability to obtain a successful COVARIANCE step, and visual inspection of basic goodness-of-fit plots.

Using fixed pharmacokinetic parameter estimates from the nintedanib base model (typical values plus empirical Bayes estimates of individual *η*), the base model of BIBF 1202 was developed. Plasma concentrations of nintedanib and BIBF 1202 were reported in nM to account for differences in their molecular weight.

### Covariate analysis

The effects of intrinsic patient factors (e.g., gender, age, body weight, ethnicity, laboratory values, and NSCLC histology) and extrinsic factors (e.g., smoking history and alcohol consumption) on pharmacokinetic parameters of nintedanib were evaluated by applying a stepwise forward inclusion/backward elimination approach. Combinations of covariates and model parameters to be tested were pre-specified based on prior knowledge, physiological plausibility, or general clinical interest (see Online Resource Table S2). Potential effects of patient population (NSCLC versus IPF) or treatment regimen (monotherapy versus combination therapy with docetaxel or pemetrexed) could not be distinguished from trials effects, and were, therefore, not re-evaluated during covariate analysis.

To assess the effect of hepatic impairment on apparent total plasma clearance (CL/F) and relative bioavailability (F1), alanine transaminase (ALT), aspartate transaminase (AST), and total bilirubin (BIL) were tested individually and in combination using an adapted liver dysfunction group (LDF) composite developed for organ dysfunction studies by the Organ Dysfunction Working Group of the National Cancer Institute [26–28]. In this classification, hepatic dysfunction is classified as mild (subdivided into two subgroups), moderate, or severe according to ALT, AST, and BIL levels relative to the upper limit of normal (ULN) (see Table 1). This categorization was preferred over the classical Child–Pugh classification [29] (based on assessments of BIL, albumin, prothrombin time, encephalopathy, and ascites) commonly used to assess surgical risk in cirrhotic patients, as it has potential disadvantages for use in oncology patients [30]. Creatinine clearance (CL_CR_) estimated by the Cockcroft–Gault equation [31] was used as surrogate for renal function. The effect of UGT1A1 polymorphisms was studied by analyzing homozygous and heterozygous genotypes separately or in combination collected in the phase II trials [11, 23].

**Table 1:** Definition of liver dysfunction groups based on adapted liver dysfunction group composite developed for organ dysfunction studies by the Organ Dysfunction Working Group of the National Cancer Institute [26–28]

Variable	Transaminase levels	Total bilirubin levels
Control	AST and ALT ≤ ULN	BIL ≤ ULN
Mild 1	AST and ALT ≤ 2.5 × ULN	BIL ≤ ULN
Mild 2	AST and ALT ≤ 10 × ULN	BIL ≤ 1.5 × ULN
Moderate	AST and ALT ≤ 10 × ULN	1.5 × ULN < BIL ≤ 3 × ULN
Severe	AST or ALT > 10 × ULN or	BIL > 3 × ULN

*ALT* alanine transaminase, *AST* aspartate transaminase, *BIL* bilirubin, *ULN* upper limit of normal

Univariate assessment of all pre-specified covariate effects using various functions (linear, hockey stick, power, or step) was initially performed to guide further covariate model building (see Online Resource Table S2). This was followed by a forward inclusion and backward elimination procedure, with significance levels of 5 and 0.1% (log-likelihood ratio test, Chi square distribution), respectively. Multiplicative covariate regression models were used to evaluate covariate combinations.

The covariate analysis for BIBF 1202 was based on the fixed pharmacokinetic parameters of the final nintedanib model and base model of BIBF 1202 and followed the same principles described for nintedanib (see Online Resource Table S3).

### Final model evaluation

The predictive performance of the base and final nintedanib models was assessed by a prediction-corrected visual predictive check (pcVPC) and prediction-corrected quantitative predictive check (pcQPC), respectively. For each, 1000 data sets were simulated using the respective nintedanib model and its parameter estimates (fixed and random effects). For each simulated data set, the same number of patients, dosing history, number of observations, sampling schedule, and covariate values as in the original data were used. Observed and simulated values were prediction-corrected using the technique of Bergstrand and colleagues [32] and were compared graphically and numerically. The final nintedanib model was further evaluated by non-parametric bootstrap analysis, in which the model was fitted to 2000 bootstrap replicates generated by resampling from the original analysis data set.

Model evaluation for BIBF 1202 was analogous to nintedanib, with the exception of the non-parametric bootstrap analysis.

Finally, after the sequential pharmacokinetic analysis for nintedanib and BIBF 1202, the parameters for the final models of nintedanib and BIBF 1202 were estimated simultaneously.

### Simulations

To illustrate individual covariate effects, the change in the median steady-state nintedanib and BIBF 1202 plasma concentration–time profiles were compared to the exposure in a typical patient. The typical patient was defined by baseline medians (continuous covariate) and modes (categorical covariate) of the respective covariates in the total analyzed population.

## Results

### Description of data set

The pharmacokinetic analysis data set comprised 1191 patients (849 NSCLC, 342 IPF) from four studies providing 5611 and 5376 nintedanib and BIBF 1202 plasma concentrations, respectively, for model development. The baseline demographic data of the patients and descriptive statistics of the tested intrinsic and extrinsic covariates are given in Table 2.

**Table 2:** Summary of baseline characteristics of trial subjects

Patient characteristics	
No. patients	1191
Age (year)	62.0 (45.0–76.0)
Weight (kg)	71.5 (50.0–100.0)
Female, *n* (%)	367 (30.8)
Ethnic origin, *n* (%)	
Caucasian	899 (75.5)
Asian^a^	283 (23.7)
Black	9 (0.8)
Smoking, *n* (%)	
Non-smoker	327 (27.5)
Ex-smoker	688 (57.8)
Current smoker	176 (14.8)
Alcohol consumption, *n* (%)	
No alcohol	701 (58.9)
Alcohol consumption should not interfere with trial participation	479 (40.2)
Alcohol consumption could interfere with trial participation	11 (0.9)
CL_CR_ (mL/min)^b^	80.8 (47.1-134.3)
Alanine transaminase (U/L)	19.0 (8.0–47.0)
Aspartate transaminase (U/L)	21.1 (11.5–42.0)
Lactate dehydrogenase (U/L)	238.0 (141.0-576.3)
Total bilirubin (µmol/L)	8.2 (3.4–15.6)
Total protein (g/L)	74.0 (64.0–86.0)
Categorization of liver dysfunction, *n* (%)	
Control	1074 (90.2)
Mild 1	104 (8.7)
Mild 2	12 (1.0)
Moderate	1 (0.1)
Severe	0 (0.0)
ECOG performance score, *n* (%)	
0	269 (22.6)
1	562 (47.2)
2	18 (1.5)
Variable	
Missing (due to IPF indication)	342 (28.7)
Indication, *n* (%)	
NSCLC	849 (71.3)
IPF	342 (28.7)
Cancer histology, *n* (%)	
NSCLC—no adenocarcinoma	274 (23.0)
NSCLC—adenocarcinoma	502 (42.1)
Patients with IPF or NSCLC of unknown histology	415 (34.8)
UGT1A1 polymorphism status^c^, *n* (%)	
UGT1A1*27	
Wild-type	198 (16.6)
Mutation	0 (0.0)
UGT1A1*60	
Wild-type	62 (5.2)
Mutation	136 (11.4)
UGT1A1*6	
Wild-type	185 (15.5)
Mutation	13 (1.1)
UGT1A1*28/*36/*37	
Wild-type	125 (10.5)
Mutation^d^	146 (12.3)
Presence of liver metastases^c^, *n* (%)	
No presence (+ IPF patients)	1038 (87.2)
Presence	145 (12.2)

*CL*_*CR*_ creatinine clearance, *ECOG* Eastern Cooperative Oncology Group, *IPF* idiopathic pulmonary fibrosis, *NSCLC* non-small-cell lung cancer. Results are presented as median (5th and 95th percentiles) unless stated otherwise

^a^Asian patients included Chinese 8.2%, Korean 5.8%, Indian 4.2%, Taiwanese 1.6%, other Asian (referring to Asians living outside China, Taiwan, India or Korea) 3.9%

^b^Calculated using the Cockcroft–Gault equation [31]

^c^Patients with missing information are not shown

^d^Includes 144 with a UGT1A1*28 mutation, none with a UGT1A1*36 mutation and 2 with a UGT1A1*37 mutation

### Final pharmacokinetic model for nintedanib

The pharmacokinetic profile of nintedanib was adequately described by a one-compartment model with first-order absorption and linear elimination. Inclusion of an absorption lag time (ALAG) was also required. The residual variability was based on log-transformed nintedanib plasma concentrations with an additive random effect model. IIV could be implemented in the nintedanib apparent volume of distribution (V_2_/F), relative bioavailability (F1), and absorption-rate constant (*k*_a_). Differences in nintedanib exposure and absorption among individual trials could not be explained by any of the investigated covariates or patient characteristics and were accounted for separately. For example, F1 was 30% higher in the NSCLC phase II [23] and LUME-Lung 2 [6] trials than in the LUME-Lung 1 [5] and IPF phase II [11] trials; *k*_a_ was 120% higher in the phase II NSCLC and IPF trials [11, 24] than in the two NSCLC Phase III trials [5, 6]. No major deviation from dose linearity was observed.

Age, body weight, ethnic origin, and smoking status were statistically significant covariates influencing nintedanib exposure (See Online Resource Table S6). F1 was affected by age and smoking status, and differed significantly between the investigated Asian subgroups. CL/F was significantly influenced by body weight.

Gender, patient population (NSCLC versus IPF; see also trial effects), NSCLC histology (adenocarcinoma versus non-adenocarcinoma), therapy regimen (monotherapy versus combination therapy with docetaxel or pemetrexed; see also trial effects), ECOG performance status, presence of liver metastases, mild or moderate renal impairment (based on CL_CR_), and lactate dehydrogenase (LDH) levels had no statistically significant impact on nintedanib pharmacokinetics. Despite limited data, there was also no substantial difference in nintedanib pharmacokinetics between Black patients (9 of the 1191 patients included), alcohol consumption (11 patients included) or UGT1A1 polymorphism status.

In patients with mild hepatic impairment at start of treatment (*n* = 116), there was a statistically non-significant trend towards lower CL/F or higher F1 values. During univariate assessment, F1 was estimated to increase by 8 and 13% for the mild 1 (*n* = 104) and mild 2 (*n* = 12) liver dysfunction categories, respectively. The limited number of patients did not allow assessment of the moderate (*n* = 1) or severe (*n* = 0) liver dysfunction categories.

Table 3 displays the final model parameter estimates for nintedanib and their precision. For a typical patient defined by the mode/median of the baseline covariate values (i.e., Caucasian, aged 62 years, weighing 71.5 kg, ex- or non-smoker) who received nintedanib, the typical CL/F was 897 L/h, the V_2_/F in the central compartment at steady state was 465 L, *k*_a_ was 0.0827 h^−1^, and ALAG was 0.417 h. Evaluation of the population mean value of half-life suggests an elimination half-life of 0.36 h (calculated as 0.693 × V_2_/CL), whereas the absorption half-life (calculated as 0.693/ka) was 8.38 h, suggesting flip-flop pharmacokinetic behavior [33], in which absorption becomes the rate-limiting metric of exposure. IIV in F1 expressed as coefficient of variation (CV) was 49.1% and IIV in V_2_/F and *k*_a_ were 119 and 32.4%, respectively. IOV in F1 of small-to-moderate extent (CV 38.9%) was detected. However, for most patients, pharmacokinetic samples were only available on two occasions and at two timepoints (one pre- and one post-dose). Considering that absorption might not follow perfect first-order kinetics, clear separation of IOV and residual variability (due to analytical precision of nintedanib measurements, imprecise sampling times, or model misspecifications) was challenging. Adding IOV to the nintedanib model only resulted in minor changes to the fixed-effect parameter estimates (including time varying covariate effects) and it was, therefore, not implemented. The η-shrinkages for IIV in F1, V_2_/F, and *k*_a_ were 13.9, 60.0, and 37.5%, respectively. The overall ε-shrinkage was 12.7%. The estimates for *k*_a_ and IIV in *k*_a_ were based on the Phase II trials [11, 23], as the absorption phase was considered to be captured better in these trials due to the higher number of available post-dose samples.

**Table 3:** Parameter estimates from the final population pharmacokinetic model of nintedanib

Parameter	Estimate (RSE%^a^)	Bootstrap analysis, 95% CI
Structural model parameters (fixed effects)		
CL/F [L/h] (*θ*_CL_)	897 (2.42)	855–941
V_2_/F [L] (*θ*_V2_)	465 (10.7)	376–569
*k*_a_ [h^−1^] (*θ*_ka_)	0.0376 (7.77)	0.0323–0.0439
ALAG [h]	0.417 (5.59)	0.351–0.463
F1 (*θ*_F1_)	1.00^b^ (–)	
Covariate effects on F1		
*θ*_Ethnicity_		
Caucasian/Black/other Asian origin	1.00^b^ (–)	–
Indian/Chinese/Taiwanese origin	1.33 (5.21)	1.19–1.47
Korean origin	0.781 (6.53)	0.690–0.893
*θ*_Smok_		
Ex-or non-smoker	1.00^b^ (–)	–
Current smoker	0.794 (4.46)	0.725–0.864
*θ*_Age_	0.00959 (16.0)	0.00635–0.0126
*θ*_Trial_		
IPF Phase II [11] and LUME-Lung 1 [5]	1.00^b^ (–)	–
NSCLC Phase II [23] and LUME-Lung 2 [6]	1.30 (3.77)	1.21–1.39
Covariate effects on CL/F		
*θ*_WT_	0.619 (16.5)	0.453–0.789
Covariate effects on *k*_a_		
*θ*_Trial_		
LUME-Lung 1 [5] and LUME-Lung 2 [6]	1.00^b^ (–)	
NSCLC Phase II [23] and IPF Phase II [11]	2.20 (8.00)	1.87–2.58
Inter-individual variability		
IIV in F1 [CV%]	49.1 (6.64^c^)	45.7–52.1
IIV in *k*_a_ for Phase II studies [CV%]	32.4 (19.2^c^)	25.2–37.8
IIV in *k*_a_ for Phase III studies [CV%]	53.8 (33.8^cd^)	0.00538–67.7^d^
IIV in V_2_/F [CV%]	119 (15.7^c^)	99.3–139
Residual unexplained variability		
Additive (SD) [nM; log scale]	0.526 (4.58^c^)	0.504–0.553

F1 = 1·*θ*_Ethnicity_·(1 + *θ*_Age_∙(AGE-62)) ·*θ*_Smok_·*θ*_Trial_·e^*η*F1^

CL/F = *θ*_CL_·(WT/71.5)^*θ*WT^

*k*_a_ = *θ*_ka_·*θ*_Trial_·e^*η*ka^

V_2_/F = *θ*_V2_·e^*η*V2^

ALAG = *θ*_ALAG_

*θ* fixed-effect parameter of interest, *ALAG* absorption lag time of nintedanib, *CI* confidence interval, *CL*/*F* apparent total body clearance for nintedanib, *CV* coefficient of variation, *ECOG* Eastern Cooperative Oncology Group, *F1* relative bioavailability for nintedanib, *IIV(η)* inter-individual variability, *IPF* idiopathic pulmonary fibrosis, *k*_*a*_ first-order absorption-rate constant for nintedanib, *nM* nanomolar (nintedanib concentration in nM = 1.853 × nintedanib concentration in ng/mL), *NSCLC* non-small-cell lung cancer, *RSE* relative standard error, *V*_*2*_/*F* apparent volume of distribution for nintedanib, *SD* Standard deviation

^a^The relative standard error as provided by NONMEM

^b^Parameters were fixed to 0 or 1 as reference values

^c^Given on the variance scale

^d^By excluding terminated bootstrap runs or runs with estimates near boundary, NONMEM and bootstrap results were congruent in estimating the imprecision measures (after exclusion of these runs, RSE% and 95% CI from bootstrapping were 37.0% and 22.6–68.0, respectively; otherwise the respective measures were 52.4% and 0.00538–67.7)

**Fig. 1:**
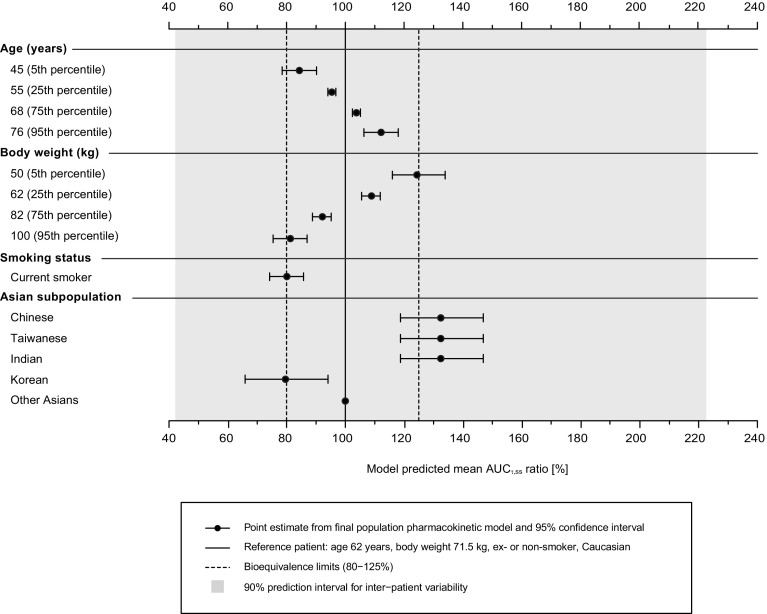
Ratios (point estimates and 95% CIs based on bootstrap analysis) of nintedanib population mean exposure (AUCτ,ss) predicted by the final model for different covariates compared with a typical patient (Caucasian, non-smoker, age 62 years, body weight 71.5 kg) receiving nintedanib treatment. The solid vertical line indicates the population mean for the typical patient, and the shaded area is the 90% prediction interval for inter-patient variability. The vertical dotted lines indicate the bioequivalence limits (80–125%). The 5th, 25th, 75th, and 95th percentiles of the baseline values observed in the analyzed population are shown for age and body weight

For the covariates age, body weight, ethnic origin, and smoking status, the model-predicted effects on the population mean nintedanib AUC_τ,ss_ are depicted in Fig. 1 and Table 4. Each of the covariates had a small-to-moderate influence on nintedanib exposure. Other than ethnic origin, the ratios of AUC_τ,ss_ for nintedanib for each factor were within the 80–125% range when varying those covariates individually within the observed extreme values (5th and 95th percentiles of baseline values for continuous covariates); exposure was increased by 33% in Chinese, Taiwanese, or Indian patients relative to a reference Caucasian patient (corrected for other covariate effects, particularly body weight).

**Table 4:** Effect of individual covariates in the final models for nintedanib and BIBF 1202 on the typical model-predicted AUC_τ,ss_ of nintedanib and BIBF 1202

Covariate	Reference patient	Percentage change in AUC_τ,ss_	
		Nintedanib	BIBF 1202
Age	62 years^a^	45 years^b^: ↓16% 76 years^c^: ↑13%	45 years^b^: ↓16% 76 years^c^: ↑13%
Smoking status	Ex- or non-smoker	Current smoker: ↓21%	Current smoker: ↓21%
Body weight	71.5 kg^a^	50 kg^b^: ↑25% 100 kg^c^: ↓19%	50 kg^b^: ↑32% 100 kg^c^: ↓22%
Ethnic origin	Caucasian	Chinese/Taiwanese/Indian: ↑33% Korean: ↓22%	Chinese/Taiwanese: ↑57% Indian: ↑141% Other Asians^d^: ↑18% Korean: ↓8%
ECOG performance status	ECOG ≥ 1	Not applicable	ECOG = 0: ↓12%
Lactate dehydrogenase	238 U/L^a^	Not applicable	141 U/L^b^: ↓8% 576 U/L^c^: ↑29%

Reference patient is Caucasian, ex- or non-smoker, age 62 years, body weight 71.5 kg, LDH level 238 U/L, ECOG performance status ≥ 1

Percentage change in AUC_τ,ss_ relative to reference patient was determined by varying values of the covariate of interest while keeping all other covariates constant. To illustrate effect size for continuous covariates, the 5th and 95th percentiles of respective baseline values were used

^a^Median of the baseline values observed in the analyzed population

^b^5th percentile

^c^95th percentile

^d^Living outside China, Taiwan, India and Korea

**Fig. 2:**
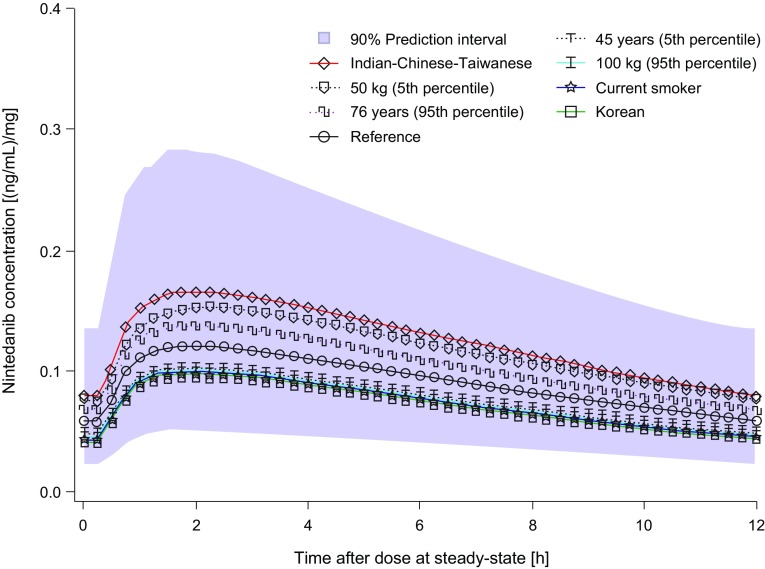
Median nintedanib plasma concentration–time profiles at steady state after multiple oral administration of nintedanib twice-daily (dose normalized) for different scenarios of covariate characteristics in relation with the median and 90% prediction interval for inter-patient variability of 2000 simulated profiles for a reference patient (Caucasian, non-smoker, age 62 years, body weight 71.5 kg). For age and body weight effects, the 5th and 95th percentiles of baseline values for the analyzed population are shown

Typical plasma profiles by subgroups of covariates with a significant influence on nintedanib plasma exposure are shown in Fig. 2. All of the median profiles based on individual covariate effects were within the 90% prediction interval profile for the typical patient.

### Final pharmacokinetic model for BIBF 1202

The pharmacokinetic profile of BIBF 1202 was best described by a one-compartment model with first-order elimination (see Online Resource Fig. S1 for model structure). To account for formation of BIBF 1202 in the intestine or via hepatic first-pass effect, a first-order absorption with lag time (ALAG_2_) was implemented as per nintedanib absorption. The relative bioavailability (F2) and the absorption-rate constant (*k*_a2_) were implemented as functions of nintedanib F1 and *k*_a_, respectively (substantial drop in OFV as compared to a model, where F2 and *k*_a2_ were estimated independently of F1 and *k*_a_). This parametrization accounted for correlations between nintedanib and BIBF 1202 parameters and replaced the full variance–covariance matrix which was not applied due to the sequential modeling approach used. The rate of formation of BIBF 1202 (*k*_met_) during nintedanib systemic elimination was accounted by defining it as a function of nintedanib clearance: *k*_met_ = CL/V_2_*ffM, where CL and V_2_ denote the clearance and central volume of distribution of nintedanib respectively, and ffM denotes the proportion of the nintedanib elimination resulting in BIBF 1202 formation. Due to the lack of data for BIBF 1202 given as an intravenous (IV) infusion, several parameter estimates were not identifiable (ffM, BIBF 1202 apparent volume of distribution [V_3_/F]) and were, therefore, fixed. The value for ffM was derived from pharmacokinetic data after IV administration of nintedanib [19] (Online Resource Table S4) and the ratio of V_3_/F–V_2_/F was fixed based on information observed in rats [14]. Despite structural identifiability of ALAG_2_, this parameter was fixed to the value of nintedanib ALAG in the final model without a substantial change in OFV (∆OFV = 1.282), as besides the comparable estimates of ALAG_2_ and ALAG, the same absorption delay for both substances was considered plausible. In addition to the (inter-individual) variability of F2 induced by the variability in F1 (F2 being proportional to F1), additional IIV could be implemented for F2.

The final model parameter estimates for BIBF 1202 are shown in Online Resource Table S5.

For BIBF 1202, the same covariates were investigated as for nintedanib. The significant covariate effects for BIBF 1202 were either induced one-to-one by a change in nintedanib F1 (age and smoking status), were influenced by a change in nintedanib pharmacokinetics but with different effect sizes for BIBF 1202 (body weight, ethnic origin, trial effects), or were identified as BIBF 1202 specific effects (ECOG performance status, LDH, and NSCLC histology) (see Online Resource Table S6).

Table 4 displays the covariates with a significant effect on BIBF 1202 exposure (AUC_τ,ss_). Besides ethnic origin, the covariates showed nearly identical effect sizes on BIBF 1202 exposure as compared to nintedanib. Additional covariates influencing BIBF 1202 exposure but not nintedanib exposure (i.e., ECOG performance status and LDH) showed only small-to-moderate changes (< 30%) in BIBF 1202 exposure. Parameter estimates for covariates not affecting BIBF 1202 exposure in terms of AUC (trial effects and NSCLC histology on *k*_a2_) are shown in Online Resource Table S5.

Median steady-state plasma concentration–time profiles for patients with covariates at the extremes of the distribution were simulated (Online Resource Fig. S2). All of the median profiles based on individual covariate effects were within the 90% prediction interval profile for the typical patient.

### Simultaneous estimation of the final nintedanib and BIBF 1202 models

Simultaneous estimation of all parameters for the final models of nintedanib and BIBF 1202 showed no major deviations of parameter estimates compared with the sequential fit (see Online Resource Table S7).

### Model evaluation and simulation

The pcVPC for the base models of nintedanib and BIBF 1202 based on the total population (Online Resource Figs. S3 and S4) showed no major discrepancies between observed and simulated concentrations after multiple nintedanib administration. In addition to pcVPC, the corresponding pcQPC for the final model focusing on the pre-dose (trough) and 2 h post-dose plasma concentration were performed. pcQPC results stratified by various covariates (Online Resource Figs. S5 and S6) show that nintedanib and BIBF 1202 exposure could be reasonably well predicted in the investigated subgroups. Only the steady-state trough levels of nintedanib at 24 h post-dose in patients with once-daily dosing were under-predicted as apparent from the pcVPC in Online Resource Fig. S3. This can be explained by a high number of samples below the limit of quantification in this subgroup affecting the 5th percentile.

The non-parametric 95% confidence intervals (2.5th and 97.5th percentiles) from bootstrap analysis for the estimates of the final nintedanib model indicated that the parameters were precisely estimated (Table 3). The estimated CVs were generally less than 20% and were congruent with the relative standard error (RSE) estimated in NONMEM. An exception was the parameter IIV in *k*_a_ of the Phase III NSCLC trials, which could not be estimated adequately (CV 52.4% by bootstrap analysis versus RSE 33.8% in NONMEM).

## Discussion

This population pharmacokinetic analysis investigated the pharmacokinetic profile of nintedanib and its main metabolite in the selected target patient populations of NSCLC and IPF. Data from 4 Phase II and III clinical trials were included. Overall, nintedanib exposure in the final model was reasonably well captured in predictive checks for the analyzed trial data and was, therefore, considered adequate for the prediction of nintedanib exposure overall and in subpopulations of covariate effects.

Nintedanib pharmacokinetic parameters were estimated by fitting a one-compartment structural model with an absorption lag, first-order input, and linear elimination to the plasma concentration versus time data. The slower rate of absorption (*k*_a_) compared with the rate of elimination (*k*_e_) suggests flip-flop pharmacokinetic behavior, where absorption is the rate-limiting process for elimination of nintedanib [33]. Though the trial data did not allow a clear differentiation between flip-flop and non-flip-flop pharmacokinetics, the flip-flop solution was pursued for the following reasons: (1) non-flip-flop-related parameter estimates could be obtained using different initial parameter values, but the OFV was consistently lower for the flip-flop solution. (2) When applying non-flip-flop kinetics in the final model, extreme estimates for V_2_ (10,700 L) and *k*_a_ (1.64 1/h for Phase II trial data) were obtained, whereas minor changes for other fixed-effect parameters (including covariate effects) were obtained. (3) After IV administration, nintedanib exhibited multi-compartmental pharmacokinetics with a very steep decline in plasma concentrations after end of infusion; the terminal half-life was, however, essentially comparable to those after oral administration indicating a redistribution-driven terminal phase. Although the terminal half-life of nintedanib should be longer following oral administration compared with IV administration for true flip-flop pharmacokinetic behavior, the nintedanib plasma profiles after oral and IV administration, nevertheless, indicate that at least some distribution processes as well as the elimination from central compartment are faster than the absorption process [19]. As a one-compartment model cannot emulate the redistribution from a (deep) peripheral compartment, the flip-flop solution was considered to be more representative and appropriate for the chosen structural model (see also Online Resource Table S4 for pharmacokinetic parameters of a one-compartment model based on data from IV administration). (4) Finally, use of a one-compartment model was considered fit for the purpose of describing steady-state (mean) exposure and assessing impact of intrinsic and extrinsic factors and follows the most parsimonious model principle supported by the data. Measures needed to enable implementation of a two- or even three-compartment model would have added complexity, which was not considered to be outweighed by the prospective benefits. Overall, the chosen modeling approach was more empirical than mechanistic (e.g., the absorption-rate limitation is not conclusively supported by the IV data). However, this approach was considered suitable for identifying subpopulations with altered nintedanib exposure and provided a valuable basis for exposure–response analyses to guide labeling recommendations.

The significantly higher nintedanib exposure observed in most of the studied Asian subpopulations countries could not be explained by differences in body weight. At present, no hypothesis can explain the underlying mechanism for the small-to-moderate increase in exposure (up to 33%) observed in the investigated Asian subpopulations. No Japanese patients were included in the trial data set.

The current analysis showed that although mild hepatic impairment, classified by elevated transaminase or bilirubin levels, showed a weak trend towards increased nintedanib exposure, it did not meet the significance criteria based on the forward inclusion–backward elimination procedure to be included in the final model. However, the limited number of patients with mild hepatic impairment (9.7% of included patients) and the lack of information about underlying hepatic disease did not allow a robust assessment of this effect. Similarly, data did not allow robust assessment of moderate hepatic impairment and there were no data for patients with severe hepatic impairment. More recent data from a dedicated hepatic impairment trial in subjects with liver cirrhosis [34] indicate an approximate 2-fold and 8-fold increases in nintedanib exposure in patients with mild or moderate hepatic impairment, categorized according to the Child–Pugh classification [29], respectively. The current model showed that patients with liver metastases (*n* = 145) had no significant effect on nintedanib pharmacokinetics. Therefore, although liver metastases alone are not considered to influence the pharmacokinetics of nintedanib if associated with other indicators of hepatic impairment, the pharmacokinetics of nintedanib can be affected. Renal function, as determined by CL_CR,_ did not have a significant impact on nintedanib pharmacokinetics after accounting for body weight. This is consistent with renal excretion having a minor role in the elimination of nintedanib.

The influence of disease (IPF versus NSCLC) or concomitant chemotherapy (docetaxel or pemetrexed) on nintedanib pharmacokinetics was assessed by accounting for potential individual trial effects. Results showed that trial specific differences in nintedanib exposure and absorption could not be explained by underlying disease or concomitant therapy. Differences in bioavailability were unlikely to be related to concomitant chemotherapy, as patients in the monotherapy trials were estimated to have the same bioavailability as those in combination therapy trials. Similarly, disease indication effects were not obvious as bioavailability in the IPF trial was estimated to be the same as that in one NSCLC trial [5]. The estimated 2-fold higher absorption-rate constant in the Phase II trials versus the Phase III trials did also not suggest an indication or comedication effect and was likely due to the higher number of post-dose samples in these trials (up to two per visit versus one per visit). The sparse pharmacokinetics sampling in the Phase III clinical trials and their timing in relation with the chemotherapy infusion (1 week apart) together with the lack of plasma exposure for combination partners did not allow a proper drug–drug interaction (DDI) assessment. Dedicated Phase I DDI trials previously showed no significant pharmacokinetic interaction between nintedanib and either docetaxel or pemetrexed [35, 36]. In this regard, this analysis provides confirmation of the Phase I DDI trial findings using longer term nintedanib treatment in the Phase III trials. Therefore, it was concluded that disease or concomitant chemotherapy did not show a systematic change in nintedanib exposure.

Despite a lower potency of BIBF 1202 exposure in in vivo assays, characterization of its pharmacokinetics, including interdependencies with the pharmacokinetics of nintedanib and identification of relevant intrinsic and extrinsic factors, was a secondary objective of this analysis, as a possible minor contribution of BIBF 1202 exposure to the overall effect in humans cannot be ruled out and effects of intrinsic and extrinsic factors could potentially cause a clinically relevant increase in BIBF 1202 exposure. Data from an earlier bioavailability trial [19] indicated a substantial formation of BIBF 1202 during first-pass metabolism. By assuming the same rate of systemic BIBF 1202 formation as estimated based on data from the previous study (fixed parameter for systemic formation rate), about 8% of BIBF 1202 in plasma was estimated to be formed systemically by the final current model.

In a dedicated absorption, distribution, metabolism, and elimination (ADME) study, the plasma concentration–time profiles of nintedanib and BIBF 1202 were similar, yielding a metabolic ratio close to 1 for AUC and *C*_max_ [16]. As the pharmacokinetics of BIBF 1202 are directly influenced by the nintedanib pharmacokinetics, the covariates influencing the parent compound indirectly also affected the pharmacokinetics of BIBF 1202. Most of the intrinsic and extrinsic factors affecting BIBF 1202 exposure did so in a manner similar to nintedanib, resulting in marginal changes in their corresponding metabolic ratio. Additional effects on BIBF 1202 exposure, particularly the higher differences in exposure for the ethnic subpopulations (e.g., a 141% higher AUC_τ,ss_ for Indian patients and a 57% higher AUC_τ,ss_ for Chinese and Taiwanese patients as compared to Caucasian patients), are not expected to change this metabolic ratio by more than 2–2.5-folds. In view of the significantly lower in vivo potency of BIBF 1202 (9-to-several hundred-folds based on cellular assays, Boehringer Ingelheim unpublished data [14]), and the close relationship between nintedanib and BIBF 1202 exposure, conclusions from the nintedanib plasma pharmacokinetic data are considered relevant for interpretation of the clinical effects of nintedanib therapy, despite a possible minor contribution of BIBF 1202 exposure to the overall effect in humans.

## Conclusion

In summary, the final model provides an adequate description of the pharmacokinetic profile of nintedanib and its main metabolite BIBF 1202 in the two target patient populations and can be used for simulations exploring covariate effects and for the development of exposure–response relationships. Patient gender, mild or moderate renal impairment, and mild hepatic impairment (classified by transaminase or bilirubin increase), underlying disease, treatment regimen, and the presence of liver metastases, had no significant effect on nintedanib exposure. The individual intrinsic and extrinsic covariates with a significant effect on nintedanib pharmacokinetics (body weight, age, smoking history, and ethnic origin) showed small-to-moderate proportional changes in exposure which individually did not exceed the observed inter-patient variability range. Similar pharmacokinetics and covariate effects were found for BIBF 1202. The results suggest that there is no need for a priori dose adjustment in the tested Asian patient subgroups (Chinese, Korean, Taiwanese, and other Asian), smokers, patients with very high or low body weight, or the young/elderly. However, particularly, a combination of equally directed covariate effects could result in more pronounced changes in systemic exposure. Due to a potentially higher frequency of adverse events, close monitoring for tolerability is warranted for patients with elevated nintedanib exposure (e.g., due to Asian race, low body weight, high age, or combinations of these risk factors).

## Electronic supplementary material

Below is the link to the electronic supplementary material.

Supplementary material 1 (DOCX 2425 KB)
