# Supplementary material for: Population pharmacokinetics of nintedanib, an inhibitor of tyrosine kinases, in patients with non-small cell lung cancer or idiopathic pulmonary fibrosis
Source: Cancer Chemother Pharmacol. 2017 Nov 8;81(1):89–101. doi: 10.1007/s00280-017-3452-0 (PMC5754397; doi:10.1007/s00280-017-3452-0)
Supplement: Supplementary file 1 — Supplementary material 1 (DOCX 2425 KB) [file 280_2017_3452_MOESM1_ESM.docx]

**Table S1** Summary of studies included in the analysis

| **Study Population** | **Therapy** | **Subjects randomized to nintedanib, n** | **Subjects included in analysis, n** | **Starting dose (mg)** | **Other doses used (mg)** | **Pharmacokinetic sampling** |
| --- | --- | --- | --- | --- | --- | --- |
| **IPF** |  |  |  |  |  |  |
| IPF; diagnosis ≤5 years prior to screening visit [1] | Monotherapy | 343 | 342 | 50 qd, 50, 100, 150 bid | 0^a^ | Pre-dose: days 1, 29, 169, 365 and any time after end of treatment  Post-dose: day 1 and day 29 at 2 (1-3) h after dosing; day 169 and day 365 at 2 (1-3) and 7 (4-10) h after dosing |
| **NSCLC** |  |  |  |  |  |  |
| Relapsed stage IIIB/IV NSCLC (after chemotherapy) [2] | Monotherapy | 73 | 73 | 250, 150 bid | 100 bid^b^ | Pre-dose: days 1, 15, 29, 43, 57, 71, 85, 106 and 127  Post-dose: day 1 and day 43 at 1, 2 and 3 h after dosing |
| Stage IIIB/IV or recurrent NSCLC after failure of first line chemotherapy [3] | Combination with 75 mg/m^2^ docetaxel every 3 weeks | 652 | 529 | 200 bid | 150, 100 bid^c^ | Pre-dose: days 29 and 50  Post-dose: day 29 at 0.5 (0.5-1) h, day 50 at 2 (1-3) h after dosing |
| Stage IIIB/IV or recurrent NSCLC after failure of first line chemotherapy [4] | Combination with 500 mg/m^2^ pemetrexed every 3 weeks | 347 | 247 | 200 bid | 150, 100 bid^c^ | Pre-dose: days 29 and 50  Post-dose: day 29 at 0.5 (0.5-1) h, day 50 at 2 (1-3) h after dosing |

*bid* twice daily, *qd* once daily, *NSCLC* non-small-cell lung cancer, *IPF* idiopathic pulmonary fibrosis

^a^ Treatment interruptions or one step dose reductions to manage adverse events were allowed (e.g. by 50 mg qd for those patients in the 50 mg qd or 50 mg bid group and by 50 mg bid for those patients in the 100 mg bid or 150 mg bid group)

^b^ Treatment interruptions or dose reductions to manage adverse events were allowed (i.e. reduction from starting dose of 250 mg bid to 150 mg bid and from 150 mg bid to 100 mg bid)

^c^ Treatment interruptions or dose reductions to manage adverse events were allowed (i.e. reduction from 200 mg bid to 150 mg bid and from 150 mg bid to 100 mg bid, when a second dose reduction was considered necessary)

**Table S2** Covariate-parameter relationships assessed for nintedanib

| **Covariate** | **F1** | **CL/F** | **V_2_/F** | **k_a_** | **Tested function** | **Rationale of covariate testing^a^** |
| --- | --- | --- | --- | --- | --- | --- |
| AGE | x | x | x | x | Linear | Physiological plausibility, trend in previous analysis [5] |
| SEX | x | x | x | x | Step function | Of general interest |
| WT | x | x | x |  | Power | Physiological plausibility, trend in previous analysis [5] |
| BMI | x | x | x |  | Linear | Physiological plausibility, trend in previous analysis [5] |
| BSA | x | x | x |  | Power | Physiological plausibility |
| SMOK | x | x | x |  | Step function | Of general interest |
| ALC | x | x | x |  | Step function | Of general interest |
| ADEN | x | x | x | x | Step function | Of general interest |
| ECOG | x | x | x |  | Step function | Of general interest |
| LMET | x | x | x |  | Step function | Physiological plausibility and trend in previous analysis [6] |
| RACE (Caucasian vs. Asian vs. Black) | x | x | x | x | Step function | Of general interest |
| ETHNIC (Asian subgroups vs. Caucasians and Blacks) | x | x | x | x | Step function | Of general interest |
| CRCL | x | x |  |  | Linear; Hockey stick | Physiological plausibility |
| ALT at baseline | x | x |  |  | Linear | Physiological plausibility and trend in previous analysis [5] |
| AST at baseline | x | x |  |  | Linear | Physiological plausibility and trend in previous analysis [5,6] |
| BIL at baseline | x | x |  |  | Linear | Physiological plausibility |
| LDF at baseline | x | x |  |  | Step function | Physiological plausibility |
| LDH | x | x | x |  | Linear; Hockey stick | Physiological plausibility and trend in previous analysis [5] |
| UGT1A1 polymorphism status | x | x |  |  | Step function | Physiological plausibility |

*ADEN* NSCLC histology (adenocarcinoma vs. non-adenocarcinoma), *AGE* age, *ALC* alcohol consumption, *ALT* alanine transaminase, *AST* aspartate transaminase, *BIL* total bilirubin, *BMI* body mass index, *BSA* body surface area, *CL/F* apparent total body clearance for nintedanib, *CRCL* creatinine clearance, *ECOG* Eastern Cooperative Oncology Group performance status, *F1* relative bioavailability for nintedanib, *k_a_* first-order absorption rate constant for nintedanib, *LDF* categorization of liver dysfunction, L*DH* lactate dehydrogenase, *RACE* race, *ETHNIC* ethnic origin, *SEX* gender, *LMET* Liver metastases, *SMOK* smoking history, *V_2_/F* apparent volume of distribution for nintedanib, WT body weight

^a^Covariate-parameter relationships were investigated based on prior knowledge (e.g. from previous covariate analyses [5,6]), physiological plausibility (referring to covariates known to potentially influence absorption, distribution or metabolism pathways relevant for nintedanib), out of general interest (referring to covariates not known to directly influence absorption, distribution or metabolism pathways relevant for nintedanib but being of general interest)

**Table S3** Covariate-parameter relationships assessed for BIBF 1202

| **Covariate*** | **F2** | **CL_2_/F** | **k_a2_** | **Tested function** |
| --- | --- | --- | --- | --- |
| AGE | x | x | x | Linear |
| SEX | x | x | x | Step function |
| WT^a^ | x | x^b^ |  | Power |
| SMOK | x | x |  | Step function |
| ALC | x | x |  | Step function |
| ADEN | x | x | x | Step function |
| ECOG | x | x |  | Step function |
| LMET | x | x |  | Step function |
| ETHNIC (Asian subgroups versus Caucasians and Blacks) | x | x | x | Step function |
| CRCL | x | x |  | Linear |
| ALT at baseline | x | x |  | Linear |
| AST at baseline | x | x |  | Linear |
| BIL at baseline | x | x |  | Linear |
| LDF at baseline | x | x |  | Step function |
| LDH | x | x |  | Linear |
| UGT1A1 polymorphism status | x | x |  | Step function |
| Trial (in contrast to nintedanib, not implemented in base model) | x | x | x | Linear |

*ADEN* NSCLC histology (adenocarcinoma vs. non-adenocarcinoma), *AGE* age, *ALC* alcohol consumption, *ALT* alanine transaminase, *AST* aspartate transaminase, *BIL* total bilirubin, *CL_2_/F* apparent total body clearance for BIBF 1202, *CRCL* creatinine clearance, *ECOG* Eastern Cooperative Oncology Group performance status, *F2* relative bioavailability for BIBF 1202, k_a2_ first-order absorption rate constant for BIBF 1202, *LDF* categorization of liver dysfunction, *LDH* lactate dehydrogenase, *ETHNIC* ethnic origin, *SEX* gender, *LMET* liver metastases, *SMOK* smoking history, *V_3_/F* apparent volume of distribution for BIBF 1202, *WT* body weight

^a^BMI and BSA not tested due to correlation with WT

^b^When WT was implemented on CL_2_/F, it was in addition tested on V_3_/F (otherwise no covariates were tested on V_3_/F)

*For rationale of covariate testing see **Table S2**

**Table S4** Structural parameters for nintedanib and BIBF 1202 from a population pharmacokinetic analysis based on healthy subjects (n=23^a^, 372 observations) after intravenous administration of nintedanib [7]

| **Parameter** | **Estimate (RSE%^a^)** |
| --- | --- |
| **Structural model parameters for nintedanib (fixed effects)** | |
| CL [L/h] | 154 (4.95) |
| V_2_ [L] | 60.9 (8.36) |
| ffM | 0.000931 (10.6) |
| CL_2_ [L/h] | 0.513 (17.0) |
| V_3_/V_2_ | 0.0185**^b^** |

*CL* total body clearance for nintedanib, *CL_2_* total body clearance for BIBF 1202, *RSE* relative standard error, *V_2_* volume of distribution for nintedanib, *V_3_* volume of distribution for BIBF 1202, *V_3_/V_2_* ratio of V_3_ to V_2_, *ffM* parameter characterizing the formation rate of BIBF 1202 (k_met_) during elimination of nintedanib (k_met_=CL/V_2_*ffM)

^a^Subjects having received single intravenous nintedanib infusion at doses of 1 mg (n=6), 3 mg (n=6) or 6 mg (n=12) in an absolute bioavailability study [7]. One subject (3 mg dose) was excluded from analysis due to increasing plasma concentrations of nintedanib after end of infusion

^b^Fixed to the ratio observed in rats ([8], study U10-2525)

**Table S5** Parameter estimates from final PopPK model of BIBF 1202

| **Parameter** | **Estimate (RSE%^a^)** | **95% CI^b^** |
| --- | --- | --- |
| **Structural model parameters (fixed effects)** | |  |
| CL_2_/F [L/h] (θ_CL2_) | 7.05 (6.37) | 6.17-7.93 |
| V_3_/V_2_ (θ_V3V2_) | 0.0185^c^ (–) | - |
| k_a2_/k_a_ (θ_ka2ka_) | 1.47 (8.16) | 1.23-1.71 |
| ALAG_2_/ALAG (θ_ALAG2ALAG_) | 1.00^d^ (–) | - |
| F_2_/F_1_ (θ_F2F1_) | 0.0110 (11.3) | 0.00856-0.0134 |
| ffM (θ_ffM_) | 0.000931^e^ (–) | - |
| **Covariate effects on F2** | |  |
| θ_WT_ | -0.848 (12.2) | -0.646 to -1.05 |
| θ_Ethnicity_ |  |  |
| Caucasian/Black origin | 1.00^d^ (–) | - |
| Indian origin | 1.90 (13.5) | 1.40-2.40 |
| Asian origin (except for Indian) | 1.20 (4.83) | 1.09-1.31 |
| θ_LDH_ (coefficient for LDH [U/L]) | 0.000656 (22.1) | 0.000372-0.000940 |
| θ_LDH___Breakpoint_ (breakpoint for LDH [U/L]) | 688 (22.0) | 391-985 |
| θ_ECOG_ |  |  |
| ECOG=0 | 1.00^d^ (–) | - |
| ECOG≥1 | 1.16 (4.16) | 1.07-1.25 |
| **Covariate effects on k_a2_** |  |  |
| θ_Trial_ |  |  |
| LUME-Lung 1 [3] and LUME-Lung 2 [4] | 1.00^d^ (–) | - |
| NSCLC Phase II [2] and IPF Phase II [1] | 0.756 (7.72) | 0.642-0.870 |
| θ_NSCLC_histology_ |  |  |
| Adenocarcinoma | 1.00^d^ (–) | - |
| Non-adenocarcinoma | 1.36 (8.90) | 1.12-1.60 |
| **Inter-individual variability** |  |  |
| IIV in F2 [CV%] | 56.7 (8.60^f^) | 51.7-61.2 |
| **Residual unexplained variability** |  |  |
| Additive (SD) [nM; log scale] | 0.546 (4.13^f^) | 0.523-0.568 |
| *θ* fixed-effect parameter of interest, *ALAG* lag time for absorption of nintedanib, *ALAG_2_* lag time for absorption of BIBF 1202, *CI* confidence interval, *CL_2_/F* apparent total body clearance for BIBF 1202, *CV* coefficient of variation, ECOG Eastern Cooperative Oncology Group, *F1* relative bioavailability for nintedanib, *F2* relative bioavailability for BIBF 1202, *F2/F1* ratio of F2 to F1, *ffM* parameter characterizing the formation rate of BIBF 1202 (k_met)_ during elimination of nintedanib (k_met_=CL/V_2_*ffM) | | |

**Table S5 cont.** Parameter estimates from the final PopPK model of BIBF 1202

| *IIV(η)* inter- individual variability, *k_a_* first-order absorption rate constant for nintedanib, k_a2_ first-order absorption rate constant for BIBF 1202, *k_a2_/k_a_* ratio of k_a2_ to k_a_, *LDH* lactate dehydrogenase, *nM* nanomolar (BIBF 1202 concentration in nM=1.903*BIBF 1202 concentration in ng/mL), *NSCLC* non-small-cell lung cancer, *RSE* relative standard error, V_2_/F apparent volume of distribution for nintedanib, V_3_/F apparent volume of distribution for BIBF 1202, *V_3_/V_2_* ratio of V_3_/F to V_2_/F, WT body weight, *SD* Standard deviation  ^a^The relative standard error as provided by NONMEM  ^b^Confidence interval estimated as (parameter estimate ± 1.96 x relative standard error) provided by NONMEM  ^c^Fixed to the ratio observed in rats ([8], study U10-2525)  ^d^Parameters were fixed to 0 or 1 as reference values  ^e^Fixed to value derived from analyzing pharmacokinetics data after intravenous administration of nintedanib to healthy volunteers (**See Table S4**)  ^f^Given on the variance scale  **Table 3** shows formulas for F1, V_2_/F, ALAG and k_a_  if LDH< θ_LDH_Breakpoint_ : F2 = F1·θ_F2F1_·(WT/71.5)^θWT^ ·θ_Ethnicity_·θ_ECOG_·(1 - θ_LDH_·(θ_LDH_breakpoint_ -LDH))·e^ηF2^; otherwise: F2 = F1·θ_F2F1_·(WT/71.5)^θWT^ ·θ_Ethnicity_ ·θ_ECOG_ ·e^ηF2^  V_3_/F = θ_V3V2_·V_2_/F  CL_2_/F = θ_CL2_  k_a2_ = k_a_·θ_ka2ka_·θ_NSCLC_histology_·θ_Trial_  ALAG_2_ = θ_ALAG2/ALAG_∙ALAG  ffM=θ_ffM_ |
| --- |

**Table S6** Changes in the objective function by removing significant covariate effects from the nintedanib or BIBF 1202 model after the backward elimination step of covariate analysis

| **Parameter** | **Covariate** | | **Changes in the objective function** |
| --- | --- | --- | --- |
| **Nintedanib model after backward elimination of covariate analysis** | | | |
| CL/F | Body weight (power function) | | 51.641 |
| F1 | Age (linear function) | | 30.480 |
| F1 | Chinese/Taiwanese/Indian ethnicity (categorical) | | 27.017 (Combination with Caucasian/Black/other Asian ethnicity) |
| F1 | Korean ethnicity (step function) | | 12.841 (Combination with Caucasian/Black/other Asian ethnicity) |
| F1 | Current smokers (step function) | | 24.247 (Combination with ex- or never smokers) |
| F1 | Studies NSCLC Phase II [2] and LUME-Lung 2 [4] (step function) | | 42.292 (Combination with IPF Phase II [1] and LUME-Lung 1 [3]) |
| k_a_ | Studies NSCLC Phase II [2] and IPF Phase II [1] (step function) | | 139.484 (Combination with LUME-Lung 1 [3] and LUME-Lung 2 [4]) |
| **BIBF 1202 model after backward elimination of covariate analysis** | | | |
| F2 | Body weight (power function) | 77.661 | |
| F2 | Indian (step function) | 20.689 (Combination with Asian ethnicities) | |
| F2 | Chinese, Korean, Taiwanese and other Asian (step function) | 13.173 (Combination with Caucasian/Black ethnicity) | |
| F2 | ECOG ≥1 (step function) | 18.518 (Combination with ECOG=0) | |
| F2 | LDH (Hockey-stick function) | 52.082 | |
| k_a2_ | Non-adenocarcinoma patients (step function) | 11.859 (Combination with adenocarcinoma patients) | |
| k_a2_ | Studies NSCLC Phase II [2] and IPF Phase II [1] (step function) | 11.536 (Combination with LUME-Lung 1 [3] and LUME-Lung 2 [4]) | |

*CL/F* apparent total body clearance for nintedanib, *CL_2_/F* apparent total body clearance for BIBF 1202, *ECOG* Eastern Cooperative Oncology Group, *F1* relative bioavailability for nintedanib, *F2* relative bioavailability for BIBF 1202, *IPF* idiopathic pulmonary fibrosis, *k_a_* first-order absorption rate constant for nintedanib, *k_a2_* first-order absorption rate constant for BIBF 1202, *LDH* lactate dehydrogenase, *NSCLC* non-small-cell lung cancer

**Table S7** Parameter estimates from simultaneous estimation for the combined nintedanib and BIBF 1202 PopPK model as compared to the sequential approach

| **Parameter** | **Simultaneous estimate (%RSE^a^)** | | **Sequential estimate (%RSE^a^)** | |
| --- | --- | --- | --- | --- |
| **Structural model parameters for nintedanib PK (fixed effects)** | | | | |
| CL/F [L/h] (θ_CL_) | | 875 (–) | | 897 (2.42) |
| V_2_/F [L] (θ_V2_) | | 617 (–) | | 465 (10.7) |
| k_a_ [h^-1^] (θ_ka_) | | 0.0391 (–) | | 0.0376 (7.77) |
| ALAG [h] | | 0.470 (–) | | 0.417 (5.59) |
| F1 (θ_F1_) | | 1.00^b^ (–) | | 1.00^b^ (–) |
| **Covariate effects on F1** | |  | |  |
| θ_Ethnicity_ | |  | |  |
| Caucasian/Black/other Asian origin | | 1.00^b^ (–) | | 1.00^b^ (–) |
| Indian/Chinese/Taiwanese origin | | 1.35 (–) | | 1.33 (5.21) |
| Korean origin | | 0.768 (–) | | 0.781 (6.53) |
| θ_Smok_ | |  | |  |
| Ex-or non-smoker | | 1.00^b^ (–) | | 1.00^b^ (–) |
| Current smoker | | 0.792 (–) | | 0.794 (4.46) |
| θ_Age_ | | 0.00942 (–) | | 0.00959 (16.0) |
| θ_Trial_ | |  | |  |
| IPF Phase II [1] and LUME-Lung 1 [3] | | 1.00^b^ (–) | | 1.00^b^ (–) |
| NSCLC Phase II [2] and LUME-Lung 2 [4] | | 1.32 (–) | | 1.30 (3.77) |
| **Covariate effects on CL/F** | |  | |  |
| θ_WT_ | | 0.601 (–) | | 0.619 (16.5) |
| **Covariate effects on k_a_** | |  | |  |
| θ_Trial_ | |  | |  |
| LUME-Lung 1 [3] and LUME-Lung 2 [4] | | 1.00^b^ (–) | | 1.00^b^ (–) |
| NSCLC Phase II [2] and IPF Phase II [1] | | 2.26 (–) | | 2.20 (8.00) |
| **Inter-individual variability for nintedanib** | |  | |  |
| IIV in F1 [CV%] | | 52.3 (–) | | 49.1 (6.64^c^) |
| IIV in k_a_ for Phase II studies [CV%] | | 35.6 (–) | | 32.4 (19.2^c^) |
| IIV in k_a_ for Phase III studies [CV%] | | 71.8 (–) | | 53.8 (33.8^cd^) |
| IIV in V_2_/F [CV%] | | 99.2 (–) | | 119 (15.7^c^) |
| **Residual unexplained variability for nintedanib** | | | | |
| Additive (SD) [nM; log scale] | | 0.506 (–) | 0.526 (4.58 ^c^) | |

**Table S7 cont.** Parameter estimates from simultaneous estimation for the combined nintedanib and BIBF 1202 PopPK model as compared to the sequential approach

| **Parameter** | **Simultaneous estimate (%RSE^a^)** | **Sequential estimate (%RSE^a^)** |
| --- | --- | --- |
| **Structural model parameters for BIBF 1202 (fixed effects)** | |  |
| CL_2_/F [L/h] (θ_CL2_) | 9.68 (–) | 7.05 (6.37) |
| V_3_/V_2_ (θ_V3V2_) | 0.0185^c^ (–) | 0.0185^c^ (–) |
| k_a2_/k_a_ (θ_ka2ka_) | 1.14 (–) | 1.47 (8.16) |
| ALAG_2_/ALAG (θ_ALAG2ALAG_) | 1.00^b^ (–) | 1.00^b^ (–) |
| F_2_/F_1_ (θ_F2F1_) | 0.0153 (–) | 0.0110 (11.3) |
| ffM (θ_ffM_) | 0.000931^d^ (–) | 0.000931^d^ (–) |
| **Covariate effects on F2** |  |  |
| θ_WT_ | -0.791 (–) | -0.848 (12.2) |
| θ_Ethnicity_ |  |  |
| Caucasian/Black origin | 1.00^b^ (–) | 1.00^b^ (–) |
| Indian origin | 1.81 (–) | 1.90 (13.5) |
| Asian origin (except for Indian) | 1.19 (–) | 1.20 (4.83) |
| θ_LDH_ (coefficient for LDH [U/L]) | 0.000640 (–) | 0.000656 (22.1) |
| θ_LDH___Breakpoint_ (breakpoint for LDH [U/L]) | 683 (–) | 688 (22.0) |
| θ_ECOG_ |  |  |
| ECOG=0 | 1.00^b^ (–) | 1.00^b^ (–) |
| ECOG≥1 | 1.13 (–) | 1.16 (4.16) |
| **Covariate effects on k_a2_** |  |  |
| θ_Trial_ |  |  |
| LUME-Lung 1 [3] and LUME-Lung 2 [4] | 1.00^b^ (–) | 1.00^b^ (–) |
| NSCLC Phase II [2] and IPF Phase II [1] | 0.864 (–) | 0.756 (7.72) |
| θ_NSCLC_histology_ |  |  |
| Adenocarcinoma | 1.00^b^ (–) | 1.00^b^ (–) |
| Non-adenocarcinoma | 1.24 (–) | 1.36 (8.90) |
| **Inter-individual variability for BIBF 1202** |  |  |
| IIV in F2 [CV%] | 47.9 (–) | 56.7 (8.60^e^) |
| **Residual unexplained variability for BIBF 1202** |  |  |
| Additive (SD) [nM; log scale] | 0.531 (–) | 0.546 (4.13^e^) |
| *θ* fixed-effect parameter of interest, *ALAG* lag time for absorption of nintedanib, *ALAG_2_* lag time for absorption of BIBF 1202, *CI* confidence interval, *CL/F* apparent total body clearance for nintedanib, *CL_2_/F* apparent total body clearance for BIBF 1202, *CV* coefficient of variation, ECOG Eastern Cooperative Oncology Group, *F1* relative bioavailability for nintedanib | | |

**Table S7 cont.** Parameter estimates from simultaneous estimation for the combined nintedanib and BIBF 1202 PopPK model as compared to the sequential approach

| *F2* relative bioavailability for BIBF 1202, *F2/F1* ratio of F2 to F1, *ffM* parameter characterizing the formation rate of BIBF 1202 (k_met)_ during elimination of nintedanib (k_met_=CL/V_2_*ffM), *IIV(η)* inter-individual variability, *k_a_* first-order absorption rate constant for nintedanib, k_a2_ first-order absorption rate constant for BIBF 1202, *k_a2_/k_a_* ratio of k_a2_ to k_a_, *LDH* lactate dehydrogenase, *nM* nanomolar (nintedanib concentration in nM=1.853*nintedanib concentration in ng/mL; BIBF 1202 concentration in nM=1.903*BIBF 1202 concentration in ng/mL), *NSCLC* non-small-cell lung cancer, *RSE* relative standard error, V_2_/F apparent volume of distribution for nintedanib, V_3_/F apparent volume of distribution for BIBF 1202, *V_3_/V_2_* ratio of V_3_/F to V_2_/F, WT body weight, *SD* Standard deviation  ^a^The relative standard error as provided by NONMEM (not available for simultaneous estimation due to aborted covariance step)  ^b^Parameters were fixed to 0 or 1 as reference values  ^c^Fixed to the ratio observed in rats ([8], study U10-2525)  ^d^Fixed to value derived from analyzing pharmacokinetics data after intravenous administration of nintedanib to healthy volunteers (**See Table S4**)  ^e^Given on the variance scale  F1 = 1·θ_Ethnicity_·(1+θ_Age_∙(AGE-62)) ·θ_Smok_·θ_Trial_·e^ηF1^  V_2_/F = θ_V2_·e^ηV2^  CL/F = θ_CL_·(WT/71.5)^θWT^  k_a_ = θ_ka_·θ_Trial_·e^ηka^  ALAG = θ_ALAG_  if LDH< θ_LDH_Breakpoint_ : F2 = F1·θ_F2F1_·(WT/71.5)^θWT^ ·θ_Ethnicity_·θ_ECOG_·(1 - θ_LDH_·(θ_LDH_breakpoint_ -LDH))·e^ηF2^_;_ otherwise: F2 = F1·θ_F2F1_·(WT/71.5)^θWT^ ·θ_Ethnicity_ ·θ_ECOG_ ·e^ηF2^  V_3_/F = θ_V3V2_·V_2_/F  CL_2_/F = θ_CL2_  k_a2_ = k_a_·θ_ka2ka_·θ_NSCLC_histology_·θ_Trial_  ALAG_2_ = θ_ALAG2/ALAG_∙ALAG  ffM=θ_ffM_ |
| --- |

**Fig. S1** Schematic illustration of the pharmacokinetic model structure of nintedanib and BIBF 1202

**
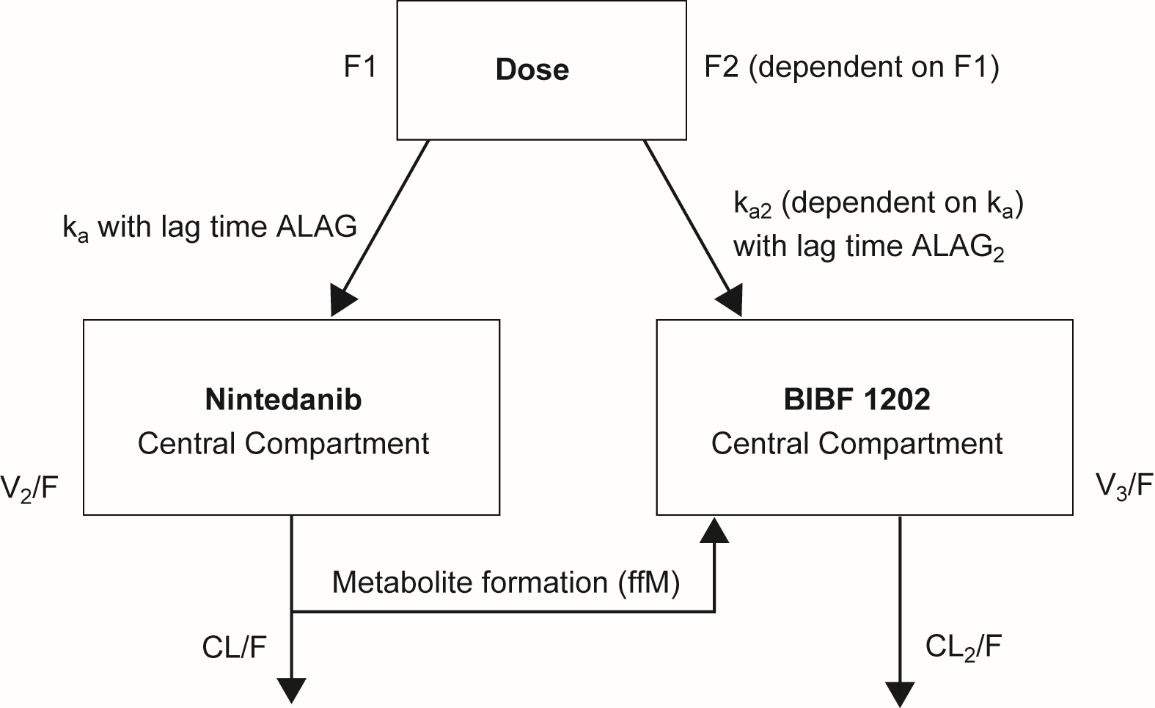
**

*CL/F* apparent total body clearance for nintedanib, *CL_2_/F* apparent total body clearance for BIBF 1202, *F1* relative bioavailability for nintedanib, *ffM* parameter characterizing the formation rate of BIBF 1202 (k_met)_ during elimination of nintedanib (k_met_=CL/V_2_*ffM), *F2* relative bioavailability for BIBF 1202, *k_a_* first-order absorption rate constant for nintedanib, *k_a2_* first-order absorption rate constant for BIBF 1202, *V_2_/F* apparent volume of distribution for nintedanib, *V_3_/F* apparent volume of distribution for BIBF 1202

**Fig. S2** Simulated median BIBF 1202 plasma concentration-time profiles at steady state after nintedanib twice-daily (dose normalized) dosing for different scenarios of covariate characteristics in relation to the median and 90% prediction interval of 2000 simulated profiles for a typical reference patient (Caucasian, non-smoker, adenocarcinoma histology, age 62 years, body weight 71.5 kg, ECOG performance score 1, LDH level 238 U/L). The solid lines show the median values and the shaded area is the 90% prediction interval of a typical patient.


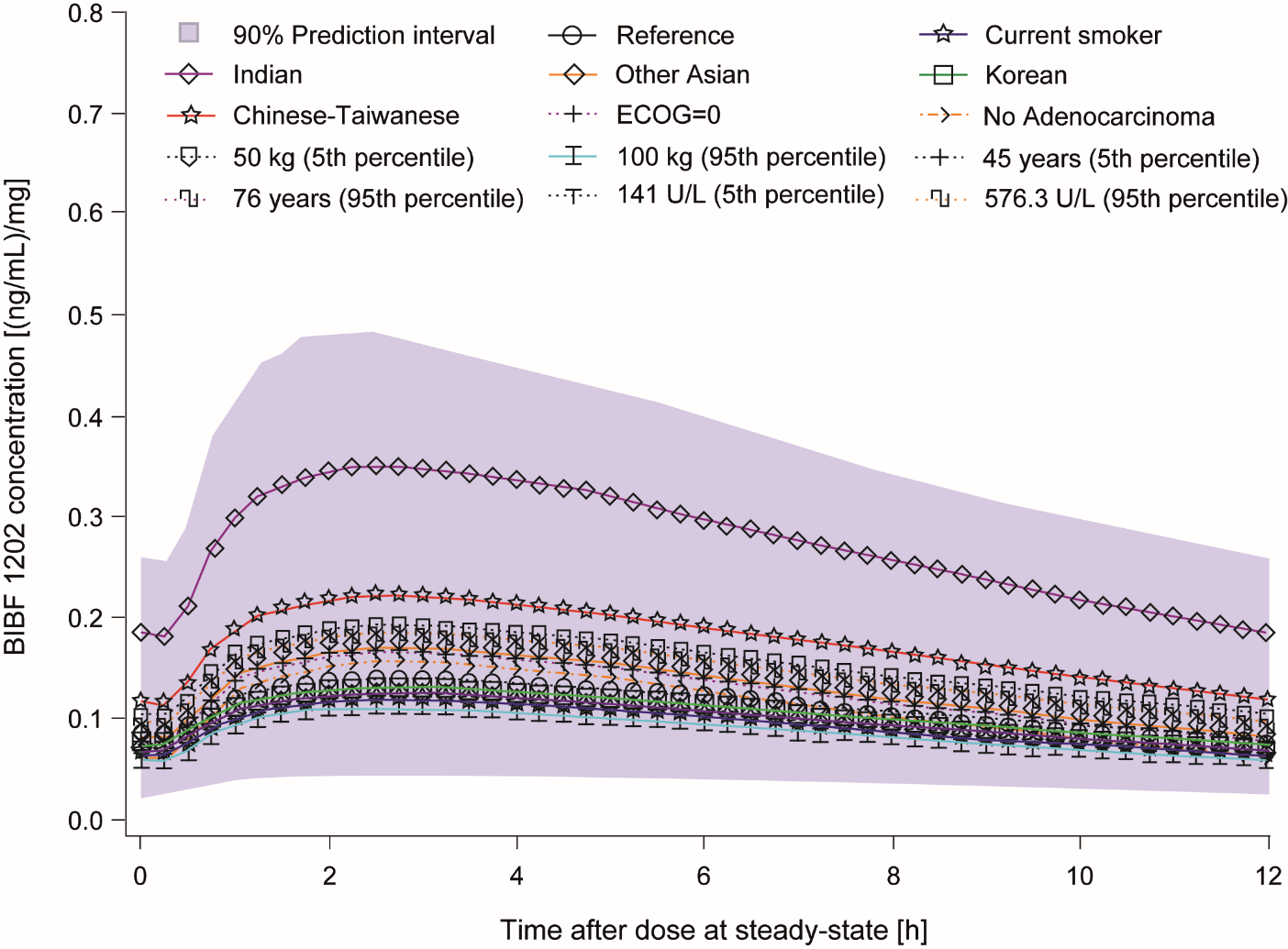


**Fig. S3** Prediction-corrected visual predictive check (pcVPC) of nintedanib plasma concentrations for the base population pharmacokinetic model across all dose levels - whole analysis population (**a** linear and **b** semi-logarithmic scale) over complete time after last dose up to 30 h. The solid lines represent the observed median and 5th and 95th percentiles of nintedanib plasma concentrations; the shaded areas are 90% prediction intervals for the median (blue) and 5th and 95th (red) percentiles of nintedanib plasma concentrations. The grey points are the individual observed nintedanib plasma concentrations up to 30 h after the last dose. In **a** the observed values >200 ng/mL are not shown (n=5).


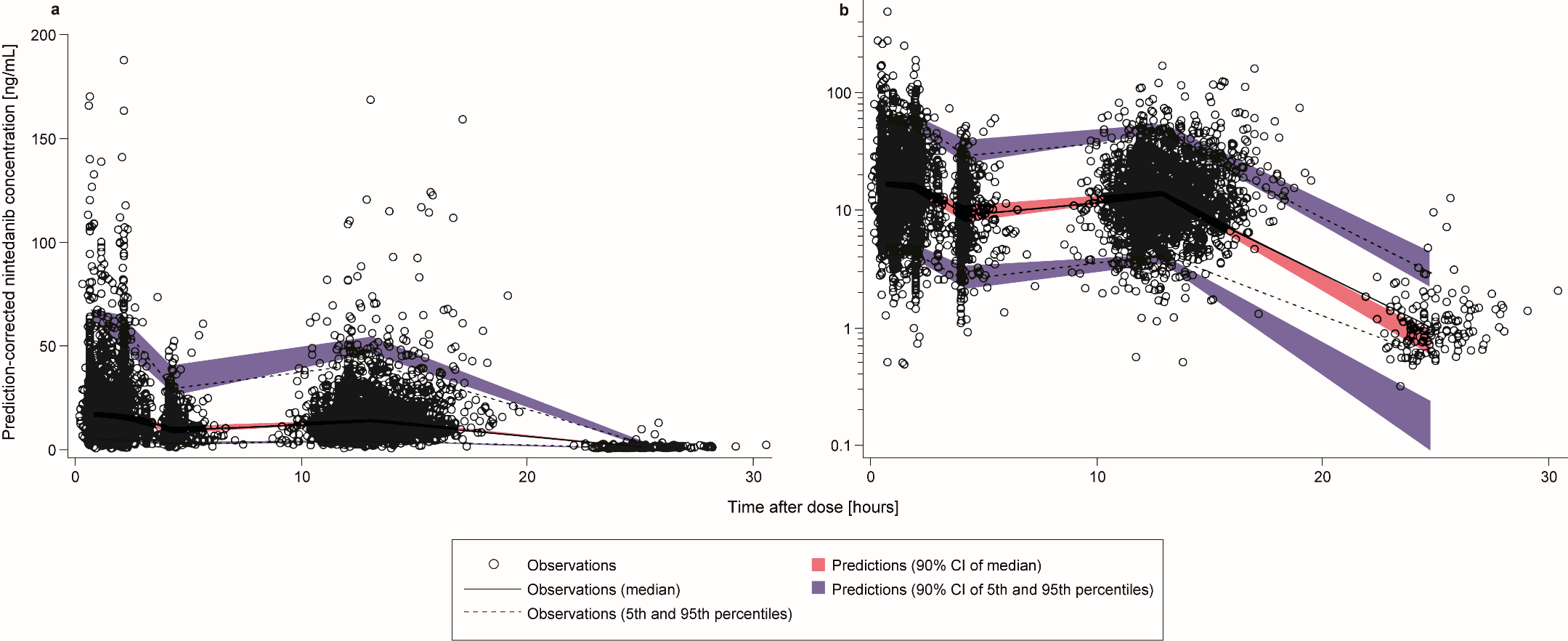


**Fig. S4** Prediction-corrected visual predictive check (pcVPC) of BIBF 1202 plasma concentrations for the base population pharmacokinetic model across all dose levels - whole analysis population (**a** linear and **b** semi-logarithmic scale) over complete time after last dose up to 30 h. The solid lines represent the observed median and 5th and 95th percentiles of BIBF 1202 plasma concentrations; the shaded areas are 90% prediction intervals for the median (blue) and 5th and 95th (red) percentiles of BIBF 1202 plasma concentrations. The grey points are the individual observed BIBF 1202 plasma concentrations up to 30 h after the last dose. In **a** the observed values >200 ng/mL are not shown (n=32).

**
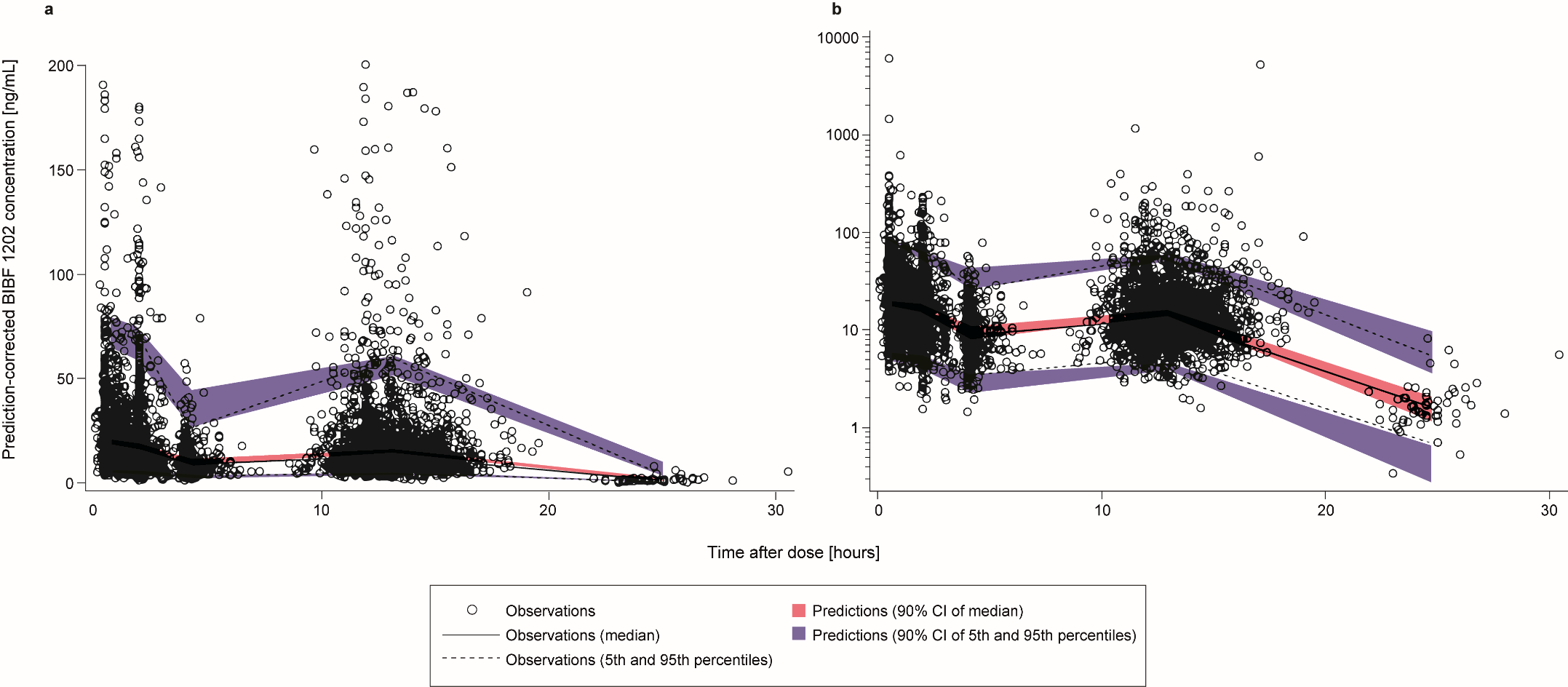
**

**Fig. S5** Prediction-corrected quantitative predictive check (pcQPC) of the final nintedanib pharmacokinetic model stratified by different covariates. Observed median steady-state trough and 2 h post-dose concentration are depicted together with corresponding simulated values (median and 95% prediction interval of all simulated median trough or 2 h post-dose).


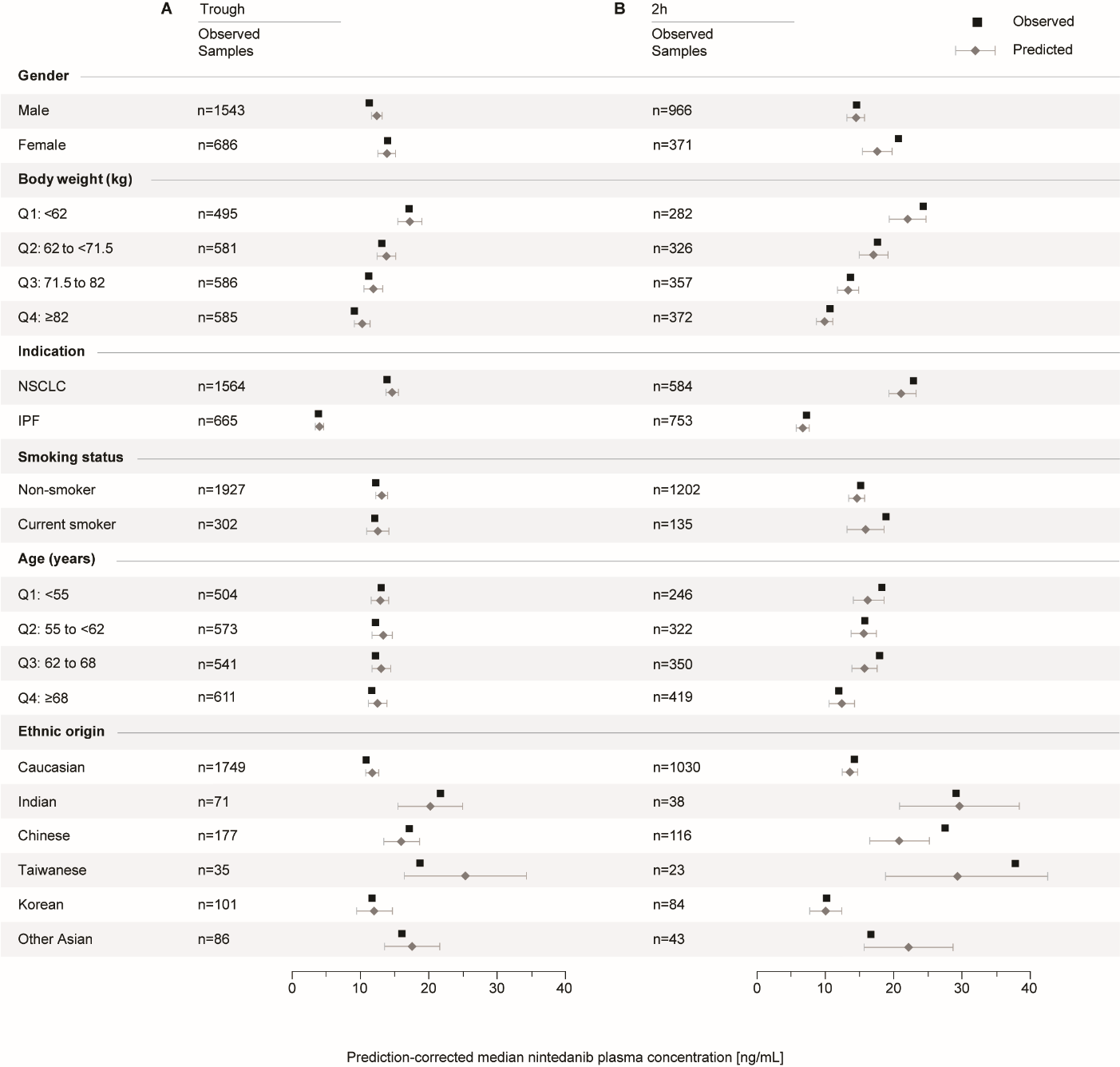


**Fig. S6** Prediction-corrected quantitative predictive check (pcQPC) of the final BIBF 1202 pharmacokinetic model stratified by different covariates. Observed median steady-state trough and 2 h post-dose concentration are depicted together with corresponding simulated values (median and 95% prediction interval of all simulated median trough or 2 h post-dose). The upper limit of the predicted 2 h post-dose concentration for Indian patients is 88 ng/mL.


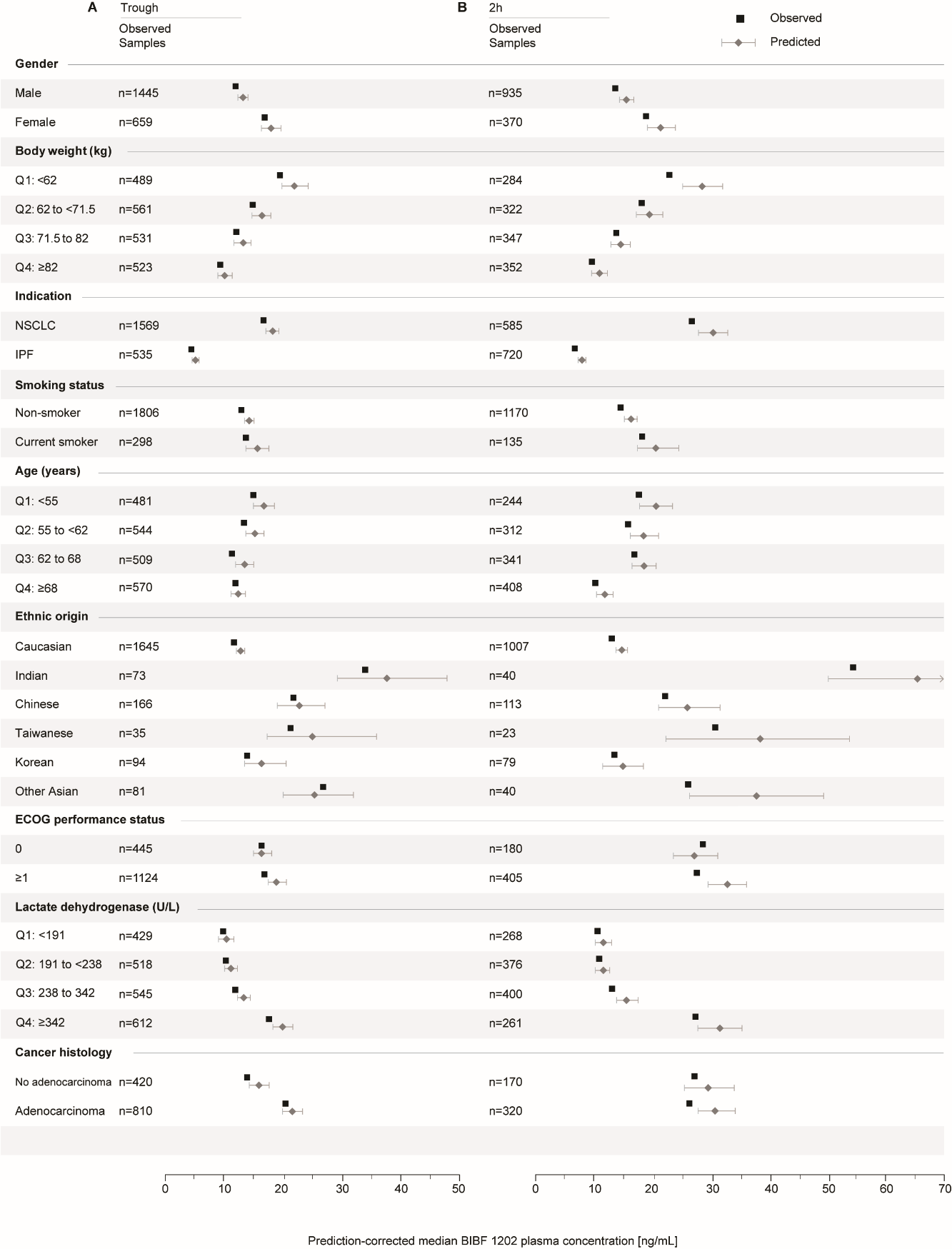


**Supplementary File Reference List**

1. Richeldi L, Costabel U, Selman M, Kim DS, Hansell DM, Nicholson AG, Brown KK, Flaherty KR, Noble PW, Raghu G, Brun M, Gupta A, Juhel N, Kluglich M, du Bois RM (2011) Efficacy of a tyrosine kinase inhibitor in idiopathic pulmonary fibrosis. N Engl J Med 365 (12):1079-1087

2. Reck M, Kaiser R, Eschbach C, Stefanic M, Love J, Gatzemeier U, Stopfer P, von Pawel J (2011) A phase II double-blind study to investigate efficacy and safety of two doses of the triple angiokinase inhibitor BIBF 1120 in patients with relapsed advanced non-small-cell lung cancer. Ann Oncol 22 (6):1374-1381

3. Reck M, Kaiser R, Mellemgaard A, Douillard JY, Orlov S, Krzakowski M, von Pawel J, Gottfried M, Bondarenko I, Liao M, Gann CN, Barrueco J, Gaschler-Markefski B, Novello S (2014) Docetaxel plus nintedanib versus docetaxel plus placebo in patients with previously treated non-small-cell lung cancer (LUME-Lung 1): a phase 3, double-blind, randomised controlled trial. Lancet Oncol 15 (2):143-155

4. Hanna NH, Kaiser R, Sullivan RN, Aren OR, Ahn MJ, Tiangco B, Voccia I, Pawel JV, Kovcin V, Agulnik J, Gaschler-Markefski B, Barrueco J, Sikken P, Schloss C, Kim JH (2016) Nintedanib plus pemetrexed versus placebo plus pemetrexed in patients with relapsed or refractory, advanced non-small cell lung cancer (LUME-Lung 2): A randomized, double-blind, phase III trial. Lung Cancer 102:65-73

5. Freiwald M, Liesenfeld K, Bruno R, Hodge L, Stopfer P, Eschbach C, Reck M, von Pawel J, Kaiser R, Staab A (2008) A population pharmacokinetic analysis for BIBF 1120, an angiokinase inhibitor, in patients with advanced non-small-cell lung cancer (NSCLC) (abstract). J Clin Oncol 26 (15_suppl):A14528

6. Stopfer P, Kaiser R, Snelder N, Ploeger B, Staab A (2006) A population pharmacokinetic model for BIBF 1120, a triple angiokinase inhibitor, in cancer patients after single and multiple oral dosing (abstract 96). EJC Suppl 4 (12):33

7. Dallinger C, Trommeshauser D, Marzin K, Liesener A, Kaiser R, Stopfer P (2016) Pharmacokinetic properties of nintedanib in healthy volunteers and patients with advanced cancer. J Clin Pharmacol 56 (11):1387-1394

8. European Medicines Agency (2014) Committee for Medicinal Products for Human Use (CHMP) Assessment Report for Ofev (nintedanib), 20 November 2014. <http://www.ema.europa.eu/docs/en_GB/document_library/EPAR_-_Public_assessment_report/human/003821/WC500182476.pdf>. Accessed May 12, 2017.
